# WTAP modulates macrophage polarization in rheumatoid arthritis by targeting exosomal circ-CBLB via m^6^A modification

**DOI:** 10.3389/fimmu.2025.1601259

**Published:** 2025-06-10

**Authors:** Dawei Yan, Lei Wan

**Affiliations:** ^1^ The First Affiliated Hospital of Anhui University of Chinese Medicine, Hefei, Anhui, China; ^2^ Center for Xin’an Medicine and Modernization of Traditional Chinese Medicine of IHM, Hefei, China; ^3^ Key Laboratory of Xin’an Medical Education Ministry, Anhui University of Chinese Medicine, Hefei, China

**Keywords:** rheumatoid arthritis, exosomes, macrophages, circ-CBLB, WTAP

## Abstract

**Introduction:**

Interactions between fibroblast-like synoviocytes (FLSs) and macrophages are pivotal in the pathogenesis of rheumatoid arthritis (RA). Exosomal circular RNAs (circRNAs) are key players in the communication between RA-FLSs and macrophages, yet their specific roles in RA pathogenesis remain undefined.This study aims to investigate the specific regulatory mechanisms of exosomal circRNAs in RA and their function in macrophage polarization through in vitro experiments.

**Methods:**

RA-FLSs were stimulated with TNF-α to mimic the enhanced inflammatory microenvironment in RA, while exosome secretion was inhibited using GW4869. WTAP expression was modulated via transfection (overexpression or knockdown), and m⁶A modification levels were analyzed using MeRIP-qPCR. Protein-RNA interactions, macrophage polarization, and cytokine profiles were evaluated through RNA pull-down assays, RIP-qPCR, flow cytometry, and ELISA, respectively.

**Results:**

In the enhanced inflammatory microenvironment of RA, circ-CBLB expression was observed to be significantly downregulated. Further functional validation showed that inhibition of exosome secretion intensified macrophage polarization toward the pro-inflammatory M1 phenotype. Screening of m^6^A modification-related enzymes combined with RNA pull-down and RIP-qPCR assays exhibited that WTAP protein directly bound to circ-CBLB and accelerated circ-CBLB degradation by enhancing its m^6^A modification levels. Functional experiments demonstrated that WTAP overexpression decreased exosomal circ-CBLB levels and promoted macrophage polarization toward the pro-inflammatory M1 phenotype, which was reversed by m^6^A modification site mutation.

**Discussion:**

This study reveals a novel mechanism that exosomal circ-CBLB secreted from RA-FLSs affects macrophage polarization under the regulation of WTAP-mediated m^6^A modification, underscoring the potential of interventions targeting the WTAP-circ-CBLB axis in RA treatment.

## 1 Introduction

Rheumatoid arthritis (RA) is an autoimmune disease characterized by chronic synovitis, progressive joint destruction, and systemic inflammation. Since the pathogenesis of the disease is complex, its pathophysiological mechanisms have not yet been fully elucidated (1, 2). In recent times, it has been increasingly unveiled that fibroblast-like synoviocytes (FLSs) and macrophage polarization occupy central roles in the pathological process of RA (3, 4). In RA, FLSs exhibit tumor-like characteristics, not only directly destroying the structure of joints by secreting matrix metalloproteinases and pro-inflammatory cytokines but also remodeling the immune microenvironment via paracrine mechanisms (5). As core effector cells in the intrinsic immune system, macrophages perform a key role in inflammatory regulation on account of their phenotypic plasticity and functional diversity (6). Macrophages can be polarized into the pro-inflammatory M1 phenotype or the anti-inflammatory M2 phenotype in response to different microenvironmental signals. M1 macrophages secrete large quantities of pro-inflammatory cytokines, such as interleukin (IL)-1β and tumor necrosis factor-alpha (TNF-α), to exacerbate tissue damage, while secreting IL-6 and chemokines, such as CCR7, to result in immune system activation, which can progress into a chronic maladaptive immune response after self-tolerance loss (7–10). M2 macrophages, instead, secrete anti-inflammatory factors, including IL-10 and transforming growth factor-beta, to control the activated immune system for tissue repair (11–13). In RA, macrophage polarization imbalance, particularly hyperactivation of M1 macrophages with inhibition of M2 macrophage function, is regarded as a key player in synovitis and bone erosion (14, 15). Nevertheless, the exact regulatory mechanisms of FLSs and macrophage polarization in RA are uncertain, which entails a great challenge for RA treatment. Consequently, it is necessary to delve deeper into the mechanisms underlying the phenotypic alterations of FLSs and the polarization of macrophages in RA.

Exosomes are small extracellular vesicles with a diameter of 30–150 nm, which are released by almost all cells under both physiological and pathological conditions (16). Importantly, exosomes harbor great potential in the treatment of inflammatory immune diseases and are central to the pathogenesis of RA (17, 18). Exosomes not only regulate immune responses by transporting cytokines but also deeply participate in the dynamic regulation of inflammatory responses by delivering biologically active molecules such as circular RNAs (circRNAs) and microRNAs (miRNAs) to trigger macrophage polarization (19, 20). Accordingly, exosomes are indispensable in immunoregulation and inflammatory regulation. CircRNAs are endogenous RNA molecules that are produced by reverse shearing of RNA (21) and can exert their function as exosomal cargoes. The biological functions of circRNAs are diverse, among which the more familiar is to act as a “sponge” for miRNAs or proteins, that is, they bind to miRNAs through complementary sequences and inhibit the regulatory effects of miRNAs on target genes, similar to the absorption of water by sponge (22). CircRNAs are highly abundant and stable because of their unique circular structure, with tremendous potential in disease diagnosis and treatment (23, 24). It has been reported that circRNAs are strongly associated with a variety of diseases, such as diabetes, cardiovascular disease, neurological disorders, and cancer (25–27). However, the role of circRNAs in the progression of RA is currently unclear compared to its role in other diseases, which calls for further in-depth studies to explore its potential role.

Our previous studies demonstrated the high value of circ-CBLB in the diagnosis and pathogenesis of RA. Nevertheless, its specific regulatory mechanism in RA is still poorly understood. In our latest study (28), it was found that circ-CBLB was significantly downregulated in RA patients and that its expression was closely related to inflammation degree and disease activity. In another study (29) involving high-throughput sequencing technology, comparisons of circRNAs and miRNAs in plasma exosomes of RA patients and healthy controls, and a rat model of adjuvant arthritis (AA), we further confirmed that circ-CBLB affected macrophage polarization through the m^6^A methylation pathway. However, little is known about the specific mechanism by which the m^6^A methylation of circ-CBLB regulates macrophage polarization. Based on the above research, we conducted an in-depth exploration around the specific mechanism of circ-CBLB in RA and innovatively proposed the hypothesis that circ-CBLB may build a bridge of pathological signaling between RA-FLSs and macrophages through exosome-mediated intercellular communication. Specifically, circ-CBLB may control the direction of macrophage polarization by activating or inhibiting downstream pathways through m^6^A methylation, thereby deeply participating in the progression of RA. Our results confirmed the above hypothesis and revealed for the first time the molecular network in which exosomal circ-CBLB derived from RA-FLSs and m^6^A methylation synergistically regulated the progression of RA. Specifically, WTAP protein overexpression in RA-FLSs accelerated the degradation of exosomal circ-CBLB through m^6^A modification, which induced macrophage polarization imbalance and potentiated RA progression. Taken together, the present study reveals a novel mechanism by which WTAP regulates macrophage polarization via m^6^A modification of RA-FLS-derived exosomal circ-CBLB, therefore providing a new perspective for RA pathogenesis and underlining the WTAP-circ-CBLB axis as a potential target for the therapeutic strategies of RA.

## 2 Materials and methods

### 2.1 Cell culture

THP-1 cells (Wuhan Pricella Biotechnology Co., Ltd., China) and RA-FLSs (Cellverse Bioscience Technology Co., Ltd., China) were resuscitated, passaged, and cryopreserved before experiments. For resuscitation, cells were obtained from liquid nitrogen, rapidly thawed in a water bath at 37°C, and then seeded for culture. During passaging, adherent RA-FLSs were passaged at 1:2 after trypsinization, while THP-1 cells were directly passaged at 1:2. THP-1 cells were seeded into 6-well plates at 1 × 10^6, treated with 100 ng/mL phorbol-12-myristate-13-acetate (PMA) overnight, and induced into M0 macrophages after 72 h. The induced M0 macrophages were co-cultured with treated RA-FLSs.

### 2.2 Media composition and passaging protocols

RA-FLSs cells were resuscitated and cultured in DMEM, whereas THP-1 cells were resuscitated and maintained in RPMI-1640. The complete DMEM medium consisted of 89% DMEM basal medium, 10% fetal bovine serum (FBS, v/v), and 1% penicillin-streptomycin double antibiotic solution (v/v). The complete RPMI-1640 medium had the same composition as DMEM complete medium, except the basal medium was replaced with RPMI-1640. The cryopreservation solution was prepared by combining 50% complete medium, 40% FBS, and 10% cell-grade dimethyl sulfoxide (DMSO, v/v). RA-FLSs cells were passaged at a 1:2 to 1:3 ratio and subcultured every 2–3 days upon reaching confluence, while THP-1 cells were passaged at a 1:2 ratio and subcultured every 2 days when confluent.

### 2.3 Treatment and grouping of RA-FLSs

After resuscitation, passaging, and cryopreservation, RA-FLSs were treated according to groups for different research purposes and then co-cultured with macrophages. As for basic treatment, some cells were treated without additional interventions as a blank control group, while some cells were treated with 20 ng/mL TNF-α for 48 h to mimic the enhanced inflammatory microenvironment in RA. To investigate the effect of exosome deficiency, 10 μM GW4869 was added to TNF-α-treated RA-FLSs (24 h) to inhibit exosome release. To clarify the function of WTAP protein, the WTAP overexpression vector was introduced into RA-FLSs using transfection reagents, with empty vectors as the negative control (NC). Small interfering RNA (siRNA) targeting WTAP was designed for transfection to knock down WTAP expression, with non-specific siRNA as the control. Finally, rescue experiments were performed to verify the role of m^6^A modification in the regulation of circ-CBLB by WTAP. Wild-type (WT) and mutant (MUT) circ-CBLB plasmids were separately transfected into cells. Additionally, these two types of plasmids were further co-transfected with WTAP overexpression vectors to observe whether the effect of WTAP on circ-CBLB was altered by m^6^A modification site mutation. Each of the above groups contained 3 biological replicates (n = 3).

### 2.4 ELISA

IL-6, TNF-α, IL-13, and IL-10 levels were measured as instructed in the manuals of corresponding ELISA kits (Ruixin Biotech, China).

### 2.5 Exosome isolation

Samples were centrifuged at 5,000 rpm for 10 min, and the supernatant was filtered through a 0.22 μm filter. EP Solution was added to the supernatant at ratios of 3:1 and 3:2, followed by 1 h of incubation at 4°C. After centrifugation at 12,000 rpm (4°C, 15 min), the supernatant was discarded. The precipitate was resuspended with 500–1,000 μL of PS Buffer through repeated pipetting, incubated at 37°C for 20 min, and further pipetted to obtain exosomes.

### 2.6 Exosome uptake assay

Exosomes were stained with the PKH26 Red Fluorescent Cell Membrane Labeling Kit (Solarbio, China). Exosome pellets were resuspended in 100 μL of 1× diluent, mixed with 2× staining solution, and incubated at 25°C for 2–5 min. THP-1 cells were seeded into 6-well plates, treated with 100 ng/mL PMA for 48 h, and incubated with labeled exosomes (90 μg/mL) for 24 h. After liquids were aspirated, cells were washed with PBS twice. Finally, images were captured under a microscope (400×).

### 2.7 Reverse transcription-quantitative polymerase chain reaction

Cell precipitates were lysed with 1 mL of TRIzol, and RNA was extracted through sequential addition of chloroform and isopropanol, centrifugation, and incubation. RNA was dissolved in DEPC water and stored. Reverse transcription was performed with a reverse transcription kit to generate cDNA, which was stored at -20°C. With the use of 2× SYBR Green qPCR Master Mix, specific primers, and cDNA, Fluorescence quantitative PCR was conducted on a StepOne Plus system (ABI, USA) using the Relative Quantification Study method. Relative expression was calculated with the 2^−ΔΔCt^ method. Primer sequences are provided in **Supplementary Materials**.

### 2.8 Methylated RNA immunoprecipitation-qPCR

RNA was extracted with the same procedure as RT-qPCR. m^6^A-containing RNA fragments were enriched with the EpiQuik CUT&RUN m6A RNA Enrichment Kit (Epigen Tek, USA). Enriched RNA was released, purified, eluted, and stored at -20°C. Reverse transcription and qPCR were carried out as described above. Primer sequences are provided in **Supplementary Materials**.

### 2.9 RNA immunoprecipitation-qPCR

RA-FLSs (2 × 10^7) were washed with PBS and lysed with polysome lysis buffer containing Protease inhibitor and RNase inhibitor. DNA was removed with DNase. Protein A/G beads were equilibrated with polysome lysis buffer. Lysates were divided into IP, IgG, and Input groups. IP and IgG samples were incubated with 3 μg of WTAP antibody (Proteintech, USA) or IgG antibody, respectively, followed by incubation with equilibrated beads. After multiple washes with Washing Buffer and DTT, RNA was eluted with polysome elution buffer, DTT, and proteinase K. Eluted RNA was extracted with TRIzol/chloroform, precipitated, and stored. Reverse transcription and qPCR were performed as described above. Primer sequences are provided in **Supplementary Materials**.

### 2.10 Flow cytometry

Cells were incubated with CD11B and CD86 antibodies (15 min, room temperature, dark), permeabilized, and then stained with CD206 antibodies (15 min, room temperature, dark). After washing, cells were resuspended in PBS containing 5% FBS, filtered using a 200 mesh filter, and analyzed on a NovoCyte flow cytometer (Agilent, USA). Data were processed using NovoExpress software.

### 2.11 Western blot

Total protein was extracted with RIPA lysis buffer containing PMSF. Proteins were separated via SDS-PAGE (5% stacking gel, 10% separating gel) and transferred to PVDF membranes. Membranes were blocked with Western blocking solutions, incubated with primary antibodies overnight, washed, and incubated with secondary antibodies. Protein bands were visualized with an automated exposure system and analyzed with Image J software. Antibody details are presented in **Supplementary Materials**.

### 2.12 RNA pull-down assay

Biotin-labeled hsa_circ-CBLB and NC probes were synthesized by BersinBio (China). Cells (2 × 10^7) were lysed with RIP buffer containing Protease inhibitor. Nucleic acids were removed using DNase. Agarose beads were pre-washed, and lysates were divided into Input, RPD, and NC groups. Biotin-labeled probes (1 μg) were denatured, ice-bathed, and folded with RNA structure buffer. Streptavidin magnetic beads were bound to RNA probes, mixed with cell lysates, RNase inhibitor, and Yeast tRNA, and washed. Proteins were eluted with Protein elution buffer and DTT. Products were analyzed with rapid silver staining and Western blot. Silver staining involved SDS-PAGE gel preparation, electrophoresis, fixation, sensitization, staining, and development.

### 2.13 Actinomycin D assay

Cells were treated with 4 μM Actinomycin D (AbMole, USA) for 0 h, 2 h, 4 h, and 8 h. RNA was extracted at each time point, and circ-CBLB expression was quantified with qPCR.

### 2.14 Cell transfection

Cells (1 × 10^6) were seeded into 6-well plates. At 70% cell confluence, siRNA-WTAP, pcDNA3.1-WTAP, WT-circ-CBLB, or MUT-circ-CBLB was mixed with Lipo8000TM transfection reagents (Beyotime, China) in serum-free medium, incubated, and added with cells. After 4 h, the medium was replaced with complete medium. Cells were harvested after 48 h for qPCR detection of transfection efficiency. For co-transfection, pcDNA3.1-WTAP/pcDNA3.1-NC was co-transfected with WT-circ-CBLB/MUT-circ-CBLB into cells as described above.

### 2.15 Immunofluorescence

Cells were permeabilized with 200 μL of Saponin (15 min, room temperature), washed with PBS, blocked with 3% BSA (20 min), and incubated with primary antibodies (4°C, overnight). Following cell incubation with secondary antibodies, nuclei were counterstained with DAPI, and slides were imaged under a fluorescence microscope. Semi-quantitative analysis was performed with Image J. Antibody details are listed in **Supplementary Materials**.

### 21.6 Statistical analysis

All data in our study were analyzed with GraphPad Prism 4.0. All experimental procedures were repeated at least 3 times. All results were presented as mean ± standard deviation unless otherwise specified. Statistical comparisons were conducted with the one-way analysis of variance or the independent samples two-tailed Student’s *t*-test. Differences at *P* < 0.05 were considered statistically significant: * *P* < 0.05, ** *P* < 0.01.

## 3 Results

### 3.1 Exosomes are released by RA-FLSs and taken up by macrophages

**Figure 1:**
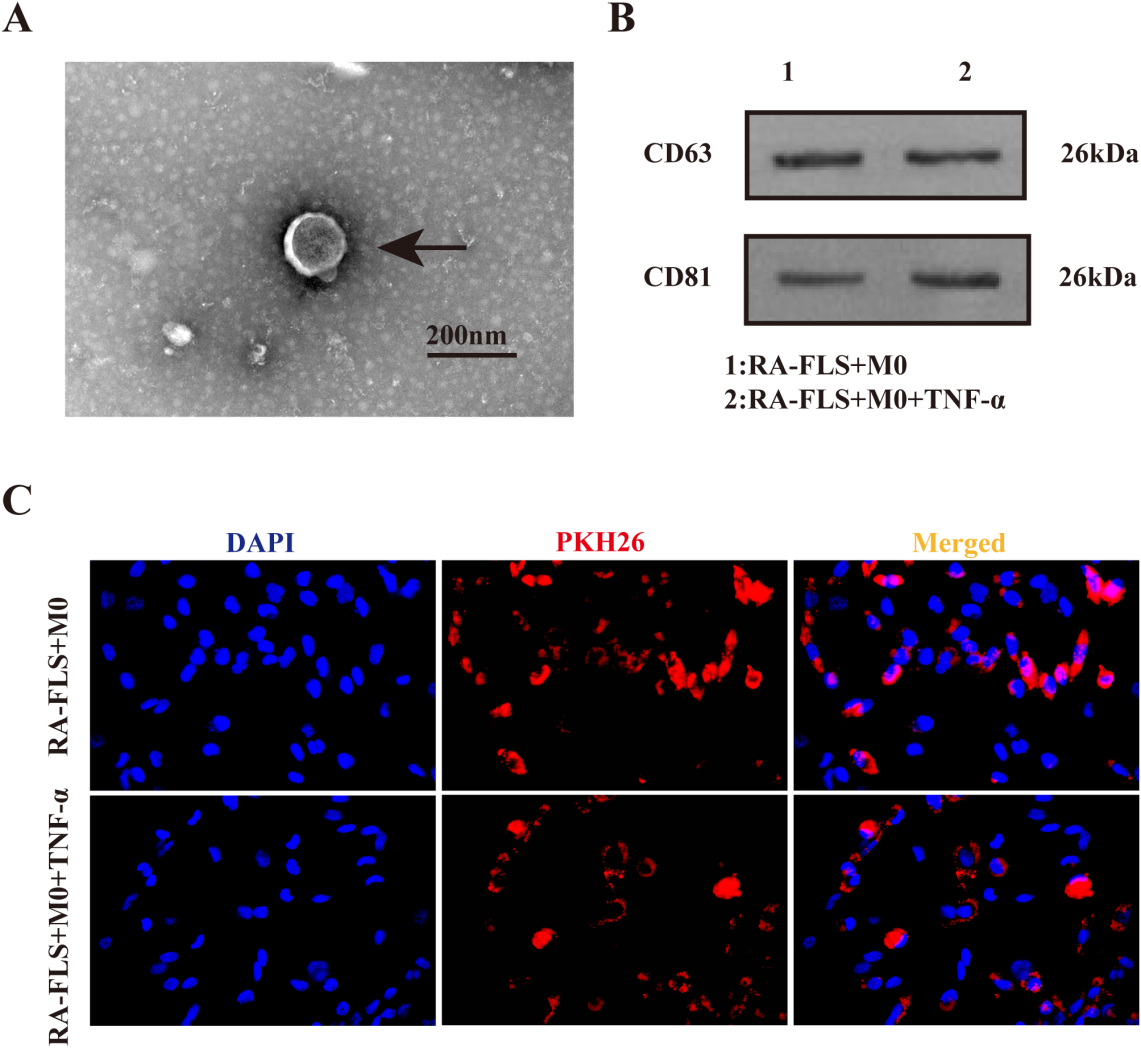
Identification of exosomes in the culture supernatant of FLSs. **(A)** Transmission electron microscopy images of exosomes. Arrows indicate typical cup-shaped exosomes. **(B)** Western blot to detect the expression of exosome-specific marker proteins CD63 and CD81. **(C)** Observation of PKH26-labeled exosomes under the fluorescence microscope.

As exhibited in **Figure 1A**, the typical cup-shaped particles with a diameter of 50−200 nm were observed under the transmission electron microscope, consistent with typical exosome features (19). Western blot demonstrated the presence of exosome-specific marker proteins CD63 and CD81 in both RA-FLS + M0 and RA-FLS + M0 + TNF-α groups (**Figure 1B**). After exosomes were labeled with PKH26, the uptake of exosomes by macrophages was successfully observed under the fluorescence microscope (**Figure 1C**).

### 3.2 Inhibition of RA-FLS-derived exosomes promotes M1 macrophage polarization

**Figure 2:**
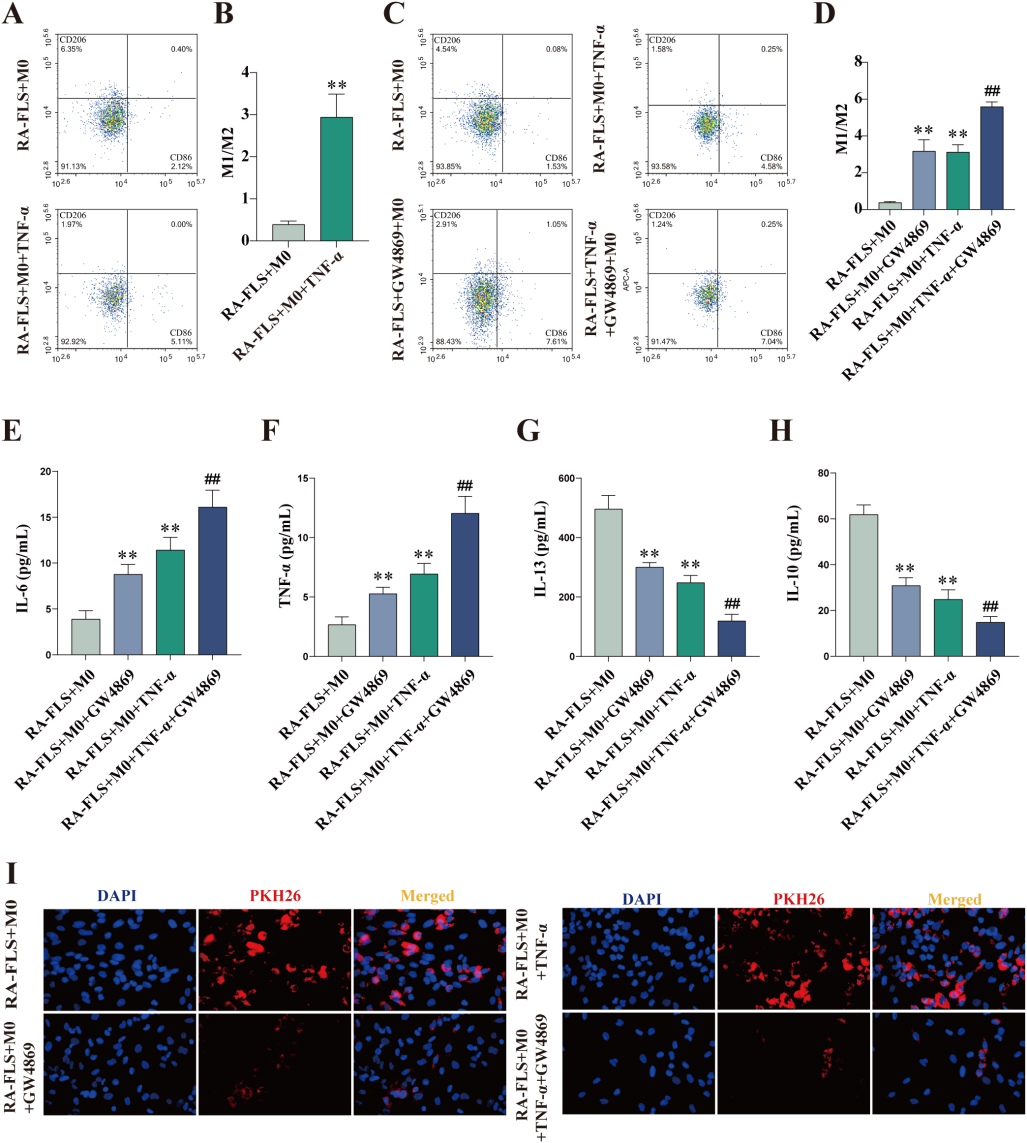
Exosome inhibition encourages macrophage polarization to the M1 phenotype. **(A, B)** Flow cytometric images of the RA-FLS + M0 and RA-FLS + M0 + TNF-α groups and quantitative analysis of the ratio of M1-type macrophages to M2-type macrophages. **(C, D)** Flow cytometric images and quantitative analysis of the ratio of M1-type macrophages to M2-type macrophages after the addition of the exosome inhibitor, GW4869. **(E-H)** ELISA to measure the levels of IL-6 **(E)**, TNF-α **(F)**, IL-13 **(G)**, and IL-10 **(E)**. **(I)** Uptake of exosomes by macrophages under the fluorescence microscope. **P < 0.01 compared with the RA-FLS + M0 group; ##*P* < 0.01 compared with the RA-FLS + M0 + TNF-α group.

CD86 and CD206 expression was measured with flow cytometry. The results showed that the M1/M2 ratio in the RA-FLS + M0 + TNF-α group was significantly higher than that in the RA-FLS + M0 group (**Figures 2A, B**). To verify whether the shift in macrophage polarization was caused by differences in exosome expression, we added the exosome inhibitor GW4869 to cells in the RA-FLS + M0 and RA-FLS + M0 + TNF-α groups, respectively, and then analyzed the expression of macrophage markers. The results revealed that the addition of GW4869 increased the M1/M2 ratio in the RA-FLS + M0 and RA-FLS + M0 + TNF-α groups (**Figures 2C, D**). The levels of IL-6, TNF-α, IL-13, and IL-10 in the supernatants of RA-FLSs co-cultured with macrophages were tested with ELISA. As found in **Figures 2E-H**, the levels of pro-inflammatory cytokines IL-6 and TNF-α were markedly higher and the levels of anti-inflammatory cytokines IL-13 and IL-10 were lower in the RA-FLS + M0 + TNF-α group than in the RA-FLS + M0 group, whereas GW4869 addition increased the levels of IL-6 and TNF-α and lowered the levels of IL-13 and IL-10 in the RA-FLS + M0 + TNF-α group. Images under the fluorescence microscope exhibited reductions in the uptake of exosomes by macrophages after the addition of GW4869 (**Figure 2I**). Taken together, macrophages were polarized toward the M1 phenotype after exosome inhibition.

### 3.3 Inhibition of exosomes reduces circ-CBLB expression

**Figure 3:**
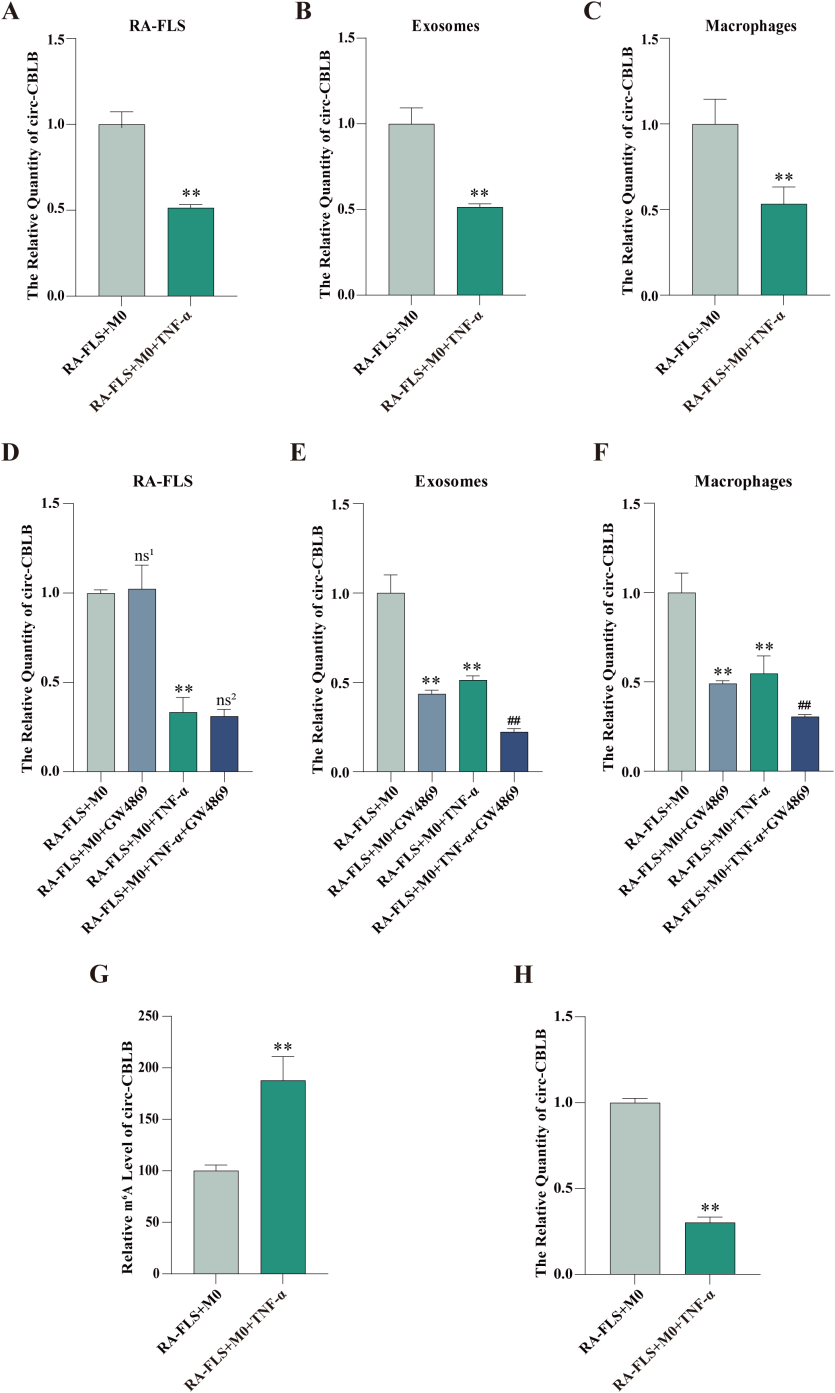
Expression and m^6^A modification levels of circ-CBLB in RA-FLSs, exosomes, and macrophages. **(A-C)** RT-qPCR detection of circ-CBLB expression in the RA-FLS + M0 and RA-FLS + M0 + TNF-α groups. **(D-F)** RT-qPCR measurement of circ-CBLB expression in RA-FLSs, exosomes, and macrophages after exosome inhibition. **(G)** MeRIP-qPCR to examine m^6^A modification levels of circ-CBLB in RA-FLSs. **(H)** RT-qPCR to determine circ-CBLB expression in RA-FLSs. **P < 0.01 compared with the RA-FLS + M0 group; ##*P* < 0.01 compared with the RA-FLS + M0 + TNF-α group; ns^1^,*P* > 0.05 compared with the RA-FLS + M0 group; ns^2^,*P* > 0.05 compared with the RA-FLS + M0 + TNF-α group.

Circ-CBLB expression in RA-FLSs, exosomes, and macrophages was examined, which displayed that circ-CBLB expression in RA-FLSs, exosomes, and macrophages was substantially lower in the RA-FLS + M0 + TNF-α group than in the RA-FLS + M0 group (**Figure 3A-C**). The addition of GW4869 did not significantly change circ-CBLB expression in RA-FLSs but declined circ-CBLB expression in exosomes and macrophages of the RA-FLS + M0 and RA-FLS + M0 + TNF-α groups (**Figures 3D-F**).

### 3.4 TNF-α stimulation diminishes circ-CBLB expression and enhances m^6^A modification in RA-FLSs

MeRIP-qPCR and RT-qPCR were used to test the m^6^A modification and expression of circ-CBLB in RA-FLSs, respectively. The results exhibited that the m^6^A modification level of circ-CBLB in the RA-FLS + M0 + TNF-α group was obviously higher than that in the RA-FLS + M0 group, accompanied by lower circ-CBLB expression (**Figures 3G, H**).

### 3.5 TNF-α stimulation elevates WTAP expression in RA-FLSs

**Figure 4:**
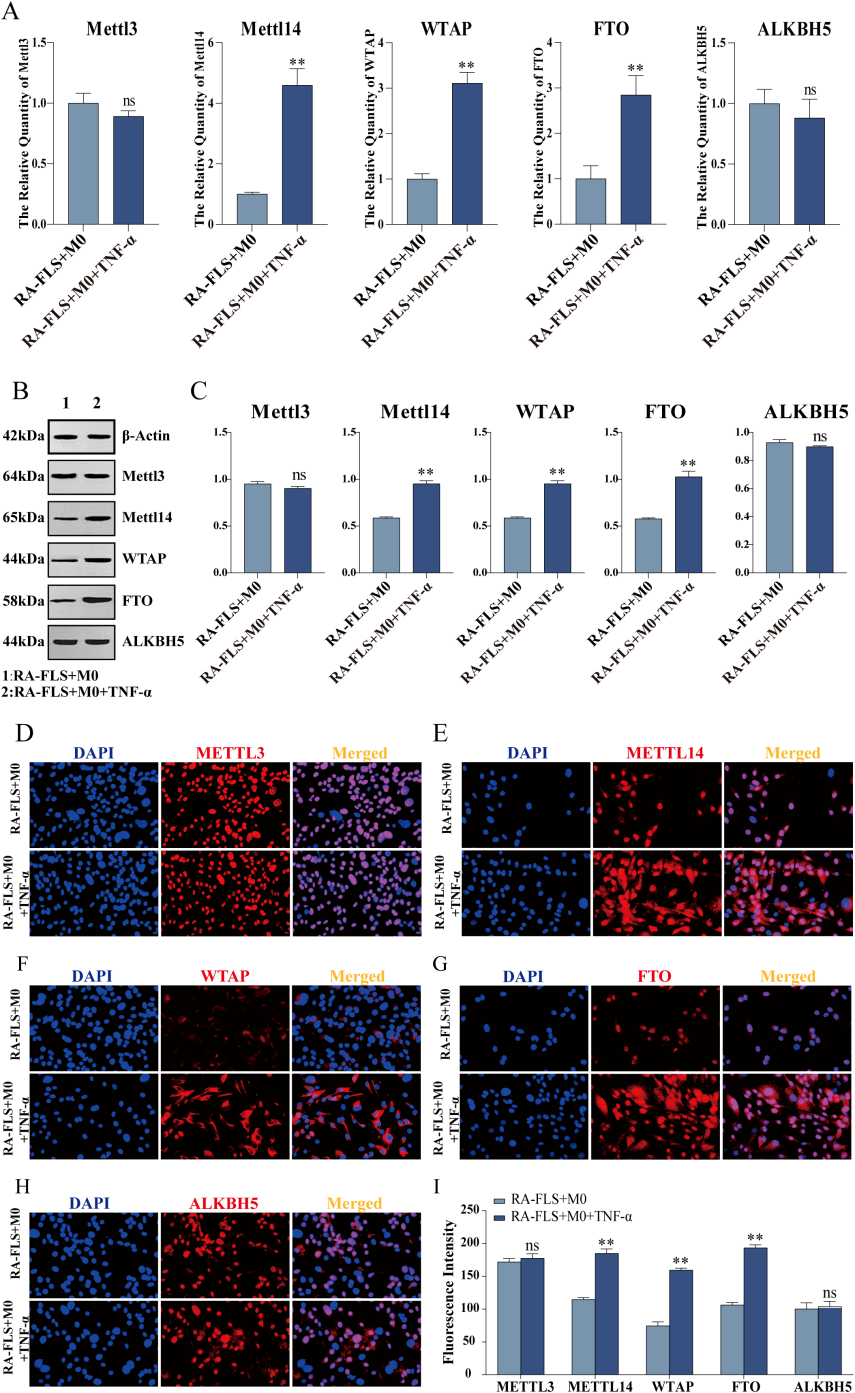
The expression of m^6^A modification-related key enzymes. **(A)** RT-qPCR to measure the mRNA expression of m^6^A modification-related key enzymes, including METTL3, METTL14, WTAP, FTO, and ALKBH5. **(B, C)** Western blot to examine the protein expression of METTL3, METTL14, WTAP, FTO, and ALKBH5 and protein concentration assay. **(D-H)** Observation of METTL3, METTL14, WTAP, FTO, and ALKBH5 expression under the fluorescence microscope. **(I)** Quantitative analysis of fluorescence intensity of METTL3, METTL14, WTAP, FTO, and ALKBH5. **P < 0.01 compared with the RA-FLS + M0 group; ns, *P* > 0.05.

RT-qPCR was performed to measure the expression of m^6^A modification-related key enzymes including METTL3, METTL14, WTAP, FTO, and ALKBH5. As depicted in **Figure 4A**, the RA-FLS + M0 + TNF-α group had prominently higher METTL14, WTAP, and FTO expression than the RA-FLS + M0 group. Further Western blot and protein concentration assay confirmed that compared with those in the RA-FLS + M0 group, RA-FLSs in the RA-FLS + M0 + TNF-α group had increased levels of METTL14, WTAP, and FTO (**Figures 4B, C**), consistent with the results of immunofluorescence (**Figures 4D-I**).

### 3.6 WTAP mutually binds to circ-CBLB

**Figure 5:**
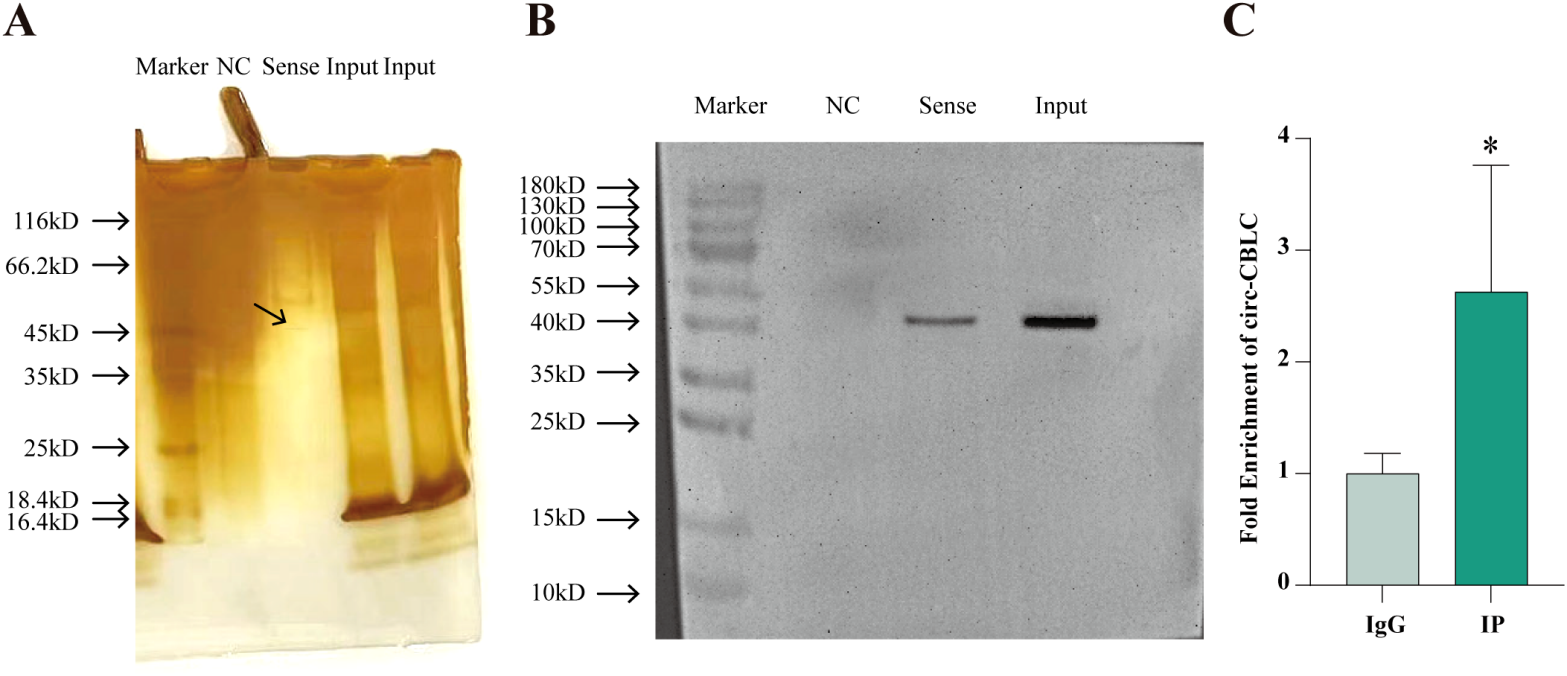
The mutual binding of WTAP and circ-CBLB. **(A)** Protein silver staining results. **(B)** Western blot to analyze the binding between circ-CBLB and WTAP. **(C)** The enrichment of circ CBLB in cells evaluated with RIP-qPCR. *P < 0.05 compared with the IgG group.

RNA pull-down and RIP assays demonstrated the mutual binding of WTAP protein and circ-CBLB (**Figure 5A, B**). The enrichment of circ-CBLB in cells was tested with RIP-qPCR, which showed a significant enrichment trend in the IP group as compared with the IgG group (**Figure 5C**).

### 3.7 WTAP overexpression increases the m^6^A modification level of circ-CBLB

**Figure 6:**
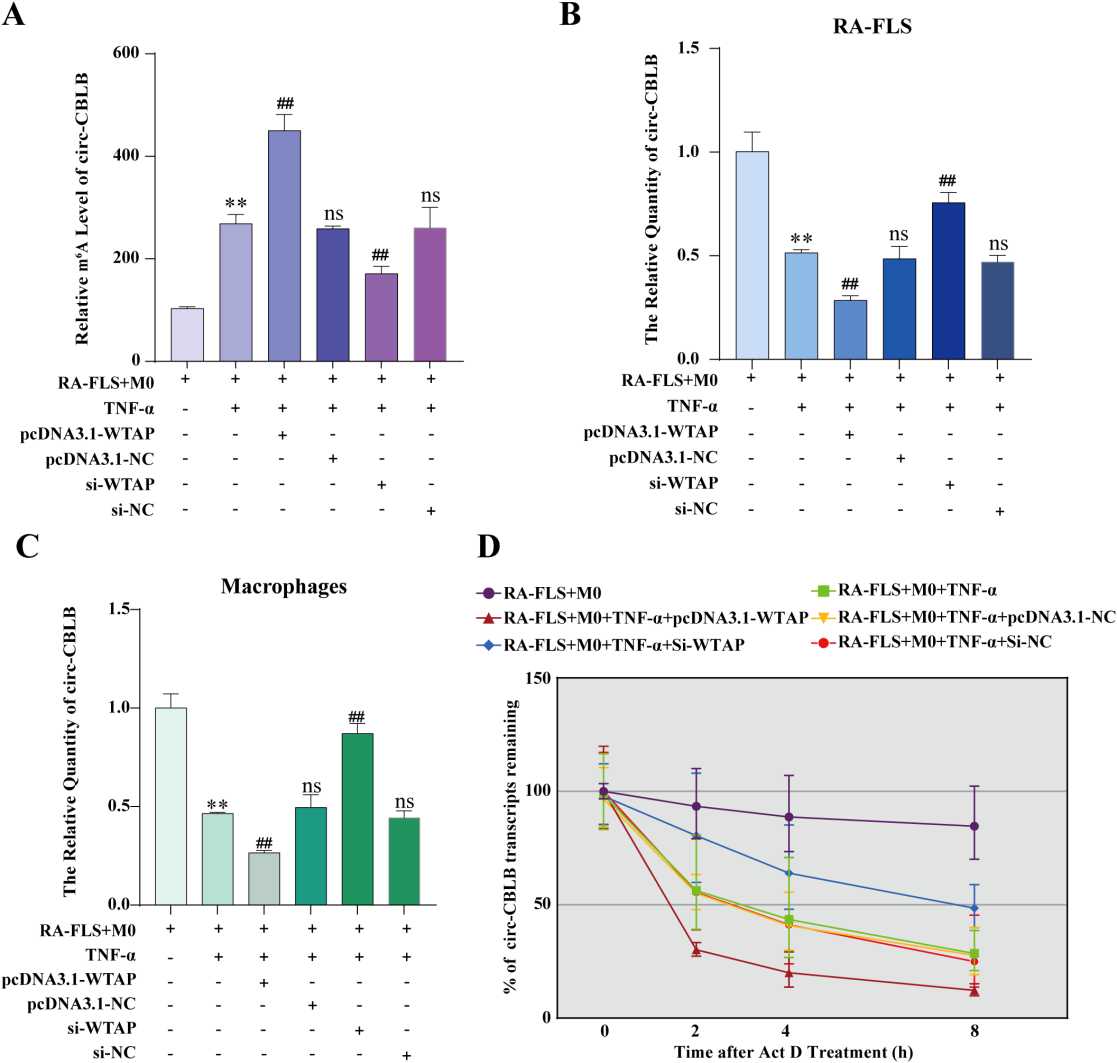
Effects of WTAP overexpression and silencing on the m^6^A modification level, expression, and half-life of circ-CBLB. **(A)** MeRIP-qPCR to analyze the m^6^A modification level of circ-CBLB in RA-FLSs. **(B, C)** RT-qPCR to detect the expression of circ-CBLB. **(D)** Half-life of circ-CBLB in RA-FLSs assessed with actinomycin D assay. **P < 0.01 compared with the RA-FLS + M0 group; ##*P* < 0.01 compared with the RA-FLS + M0 + TNF-α group; ns, *P* > 0.05 compared with the RA-FLS + M0 + TNF-α group.

To ascertain the effect of WTAP on macrophage polarization via the m^6^A modification of circ-CBLB in RA-FLSs, we constructed WTAP silencing and overexpression models and detected the m^6^A modification level of circ-CBLB in RA-FLSs using MeRIP-qPCR. The results displayed significantly higher m^6^A modification levels in the RA-FLS + M0 + TNF-α group than in the RA-FLS + M0 group. m^6^A modification levels were insignificantly different among the RA-FLS + M0 + TNF-α + pcDNA3.1-NC, RA-FLS + M0 + TNF-α + si-NC, and RA-FLS + M0 + TNF-α groups. m^6^A modification levels was markedly higher in the RA-FLS + M0 + TNF-α + pcDNA3.1-WTAP group but lower in the RA-FLS + M0 + TNF-α + si-WTAP group than in the RA-FLS + M0 + TNF-α group (**Figure 6A**). Altogether, WTAP overexpression enhanced the m^6^A modification level of circ-CBLB in RA-FLSs.

### 3.8 WTAP overexpression reduces circ-CBLB expression

According to RT-qPCR data, there was substantially lower circ-CBLB expression in the RA-FLS + M0 + TNF-α group than in the RA-FLS + M0 group, with no differences in circ-CBLB expression among the RA-FLS + M0 + TNF-α + pcDNA3.1-NC, RA-FLS + M0 + TNF-α + si-NC, and RA-FLS + M0 + TNF-α groups. Compared to the RA-FLS + M0 + TNF-α group, the RA-FLS + M0 + TNF-α + pcDNA3.1-WTAP group had lower circ-CBLB expression, whilst the RA-FLS + M0 + TNF-α + si-WTAP group had higher circ-CBLB expression (**Figure 6B**). The expression of circ-CBLB in macrophages was concordant with that in RA-FLSs (**Figure 6C**). Collectively, overexpression of WTAP decreased the expression of circ-CBLB.

### 3.9 WTAP overexpression accelerates the degradation of circ-CBLB

To further delve into the specific mechanism by which WTAP affects circ-CBLB expression, we examined the half-life of circ-CBLB in RA-FLSs under WTAP silencing or overexpression using the actinomycin D assay. As observed in **Figure 6D**, the decay rate of circ-CBLB was the lowest in the RA-FLS + M0 group and the highest in the RA-FLS + M0 + TNF-α + pcDNA3.1-WTAP group, and there were no differences in the decay rate among the RA-FLS + M0 + TNF-α + pcDNA3.1-NC, RA-FLS + M0 + TNF-α + si-NC, and RA-FLS + M0 + TNF-α groups. The decay rate of the RA-FLS + M0 + TNF-α + si-WTAP group was higher than that of the RA-FLS + M0 group and lower than that of the RA-FLS + M0 + TNF-α group.

### 3.10 WTAP overexpression drives the polarization of M0 macrophages to the M1 phenotype

**Figure 7:**
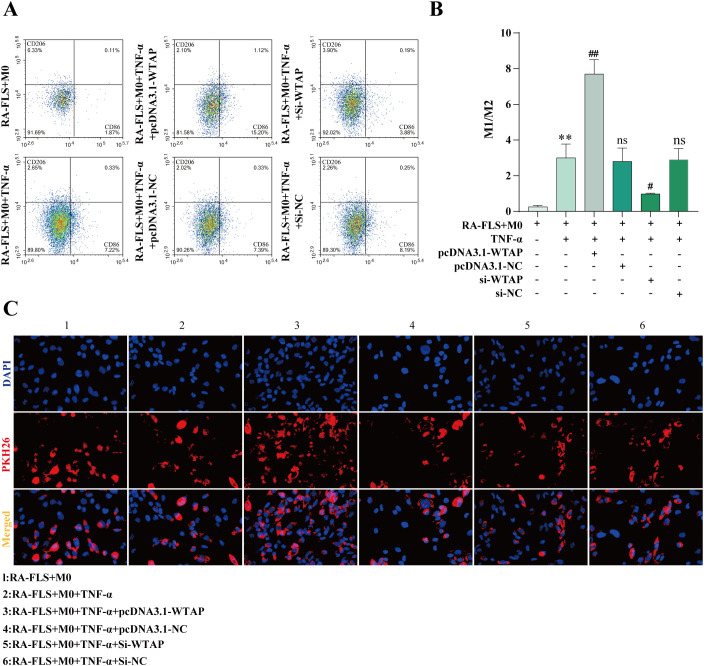
Effects of WTAP overexpression and silencing on macrophage polarization. **(A, B)** Flow cytometric images of macrophages and quantitative analysis of the ratio of M1-type macrophages to M2-type macrophages after WTAP overexpression and silencing. **(C)** Observation of exosome uptake by macrophages under the fluorescence microscope. **P < 0.01 compared with the RA-FLS + M0 group; ##*P* < 0.01 compared with the RA-FLS + M0 + TNF-α group; ns, *P* > 0.05 compared with the RA-FLS + M0 + TNF-α group.

According to flow cytometry results, M1 polarization of macrophages was enhanced in the RA-FLS + M0 + TNF-α + pcDNA3.1-WTAP group and repressed in the RA-FLS + M0 + TNF-α + si-WTAP group (**Figures 7A, B**). PKH26 labeling of exosomes demonstrated that exosomes were taken up by macrophages (**Figure 7C**).

### 3.11 Rescue Experiments Validate That m^6^A Modification Regulates circ-CBLB Expression and Stability via WTAP

**Figure 8:**
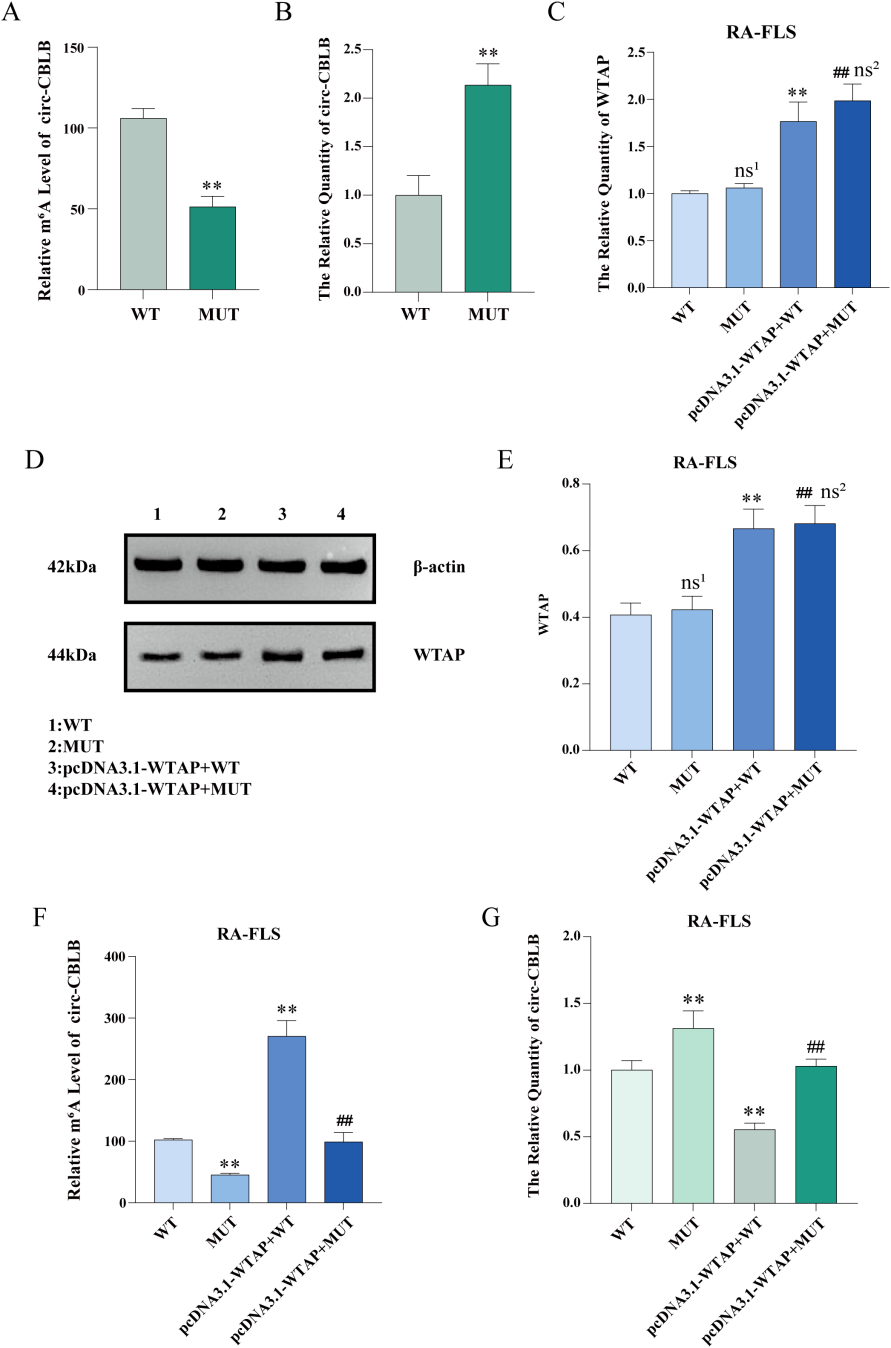
Levels of m^6^A modification, circ-CBLB, and WTAP after mutation of the m^6^A modification site. **(A)** MeRIP-qPCR detection of m^6^A modification levels of circ-CBLB in RA-FLSs before and after mutation. **(B)** RT-qPCR to measure the expression of circ-CBLB in RA-FLSs before and after mutation. **(C)** RT-qPCR to test the expression of WTAP in RA-FLSs. **(D, E)** Western blot and protein concentration assay to analyze the protein expression of WTAP in RA-FLSs. **(F)** MeRIP-qPCR to examine the m^6^A modification level of circ-CBLB in RA-FLSs. **(G)** RT-qPCR to measure the expression of circ-CBLB in RA-FLSs. **P < 0.01 compared with the WT group; ##*P* < 0.01 compared with the MUT group; ns^1^, *P* > 0.05 compared with the WT group. ns^2^, *P* > 0.05 compared with the pcDNA3.1-WTAP + WT group.

MeRIP-qPCR and RT-qPCR were used to examine the m^6^A modification level and expression of circ-CBLB in RA-FLSs, respectively. The results uncovered that the m^6^A modification level of circ-CBLB was decreased and the expression of circ-CBLB was increased in the RA-FLS + TNF-α + M0 + pcDNA3.1-NC + MUT-circ-CBLB group (the MUT group) versus the RA-FLS + TNF-α + M0 + pcDNA3.1-NC + WT-circ-CBLB group (the WT group) (**Figures 8A, B**).

### 3.12 WTAP overexpression elevates the m^6^A modification level of circ-CBLB and lowers its expression

The total RNA of RA-FLSs was extracted with TRIzol, and WTAP expression was tested with RT-qPCR. As exhibited in **Figure 8C**, no significant difference was observed in WTAP expression between the WT and MUT groups, and WTAP expression was substantially higher in RA-FLS + TNF-α + M0 + pcDNA3.1-WTAP + WT-circ-CBLB (the pcDNA3.1-WTAP + WT group) and RA-FLS + TNF-α + M0 + pcDNA3.1-WTAP + MUT-circ-CBLB groups (the pcDNA3.1-WTAP + MUT group) than in the WT and MUT groups, respectively. Western blot results were similar to the results of RT-qPCR (**Figures 8D, E**). According to MeRIP-qPCR results, the m^6^A modification level was lower in the MUT group than in the WT group, higher in the pcDNA3.1-WTAP + WT group than in the WT group, and higher in the pcDNA3.1-WTAP + MUT group than in the MUT group (**Figure 8F**). RT-qPCR results showed that circ-CBLB expression in RA-FLSs was higher in the MUT group than in the WT group, whilst circ-CBLB expression was lower in the pcDNA3.1-WTAP + WT and pcDNA3.1-WTAP + MUT groups when compared with the WT and MUT groups, respectively (**Figure 8G**).

### 3.13 WTAP overexpression potentiates the M1 polarization of macrophages

**Figure 9:**
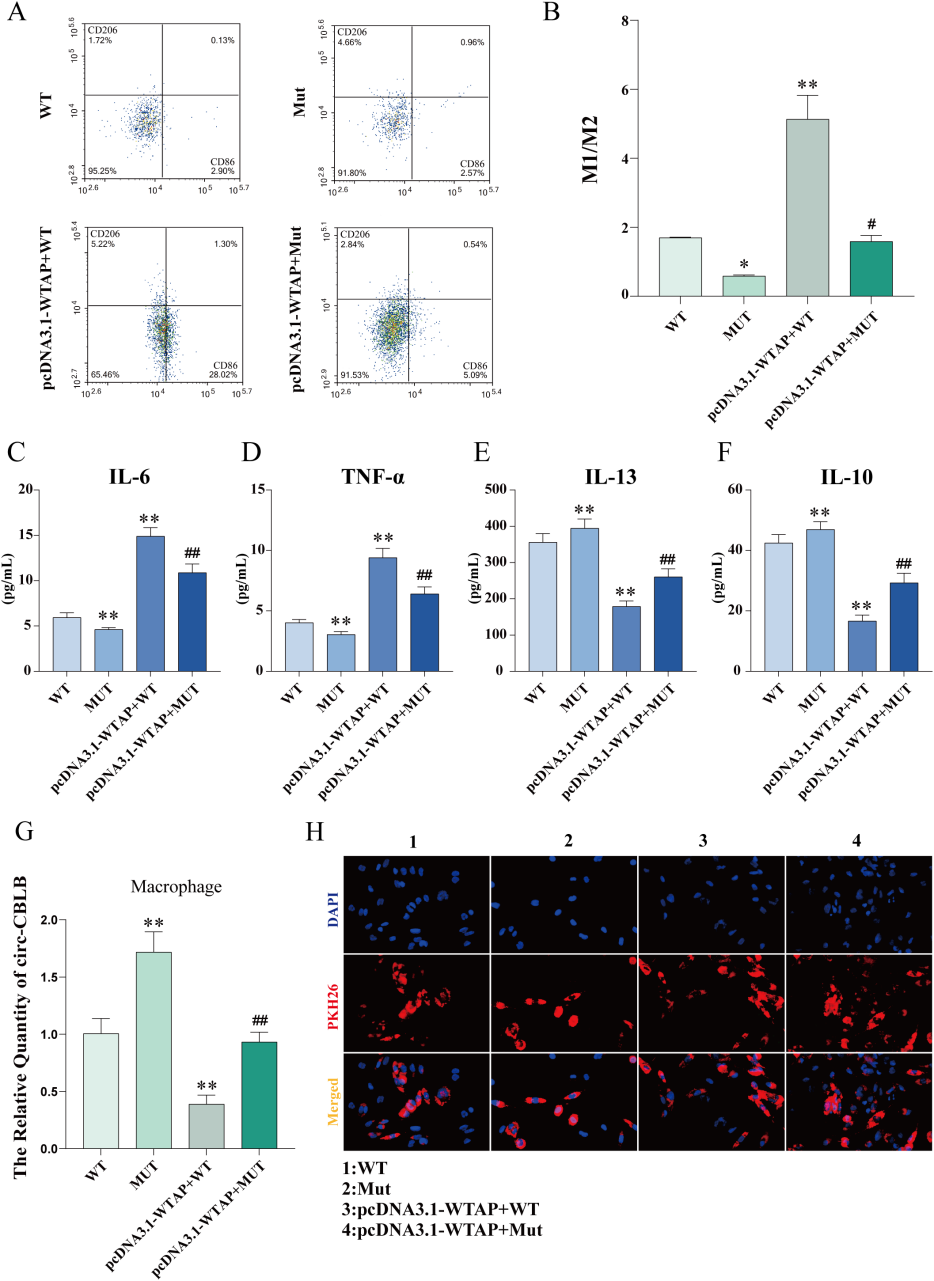
Macrophage polarization and cytokine levels after m^6^A modification site mutation. **(A, B)** Flow cytometric images of macrophages and quantitative analysis of the ratio of M1-type macrophages to M2-type macrophages after m^6^A modification site mutation. **(C-F)** ELISA to determine levels of IL-6, TNF-α, IL-13, and IL-10 in the supernatant of macrophages co-cultured with RA-FLSs after m^6^A modification site mutation. **(G)** Expression of circ-CBLB in macrophages detected with RT-qPCR after m^6^A modification site mutation. **(H)** Fluorescence microscopy to observe the uptake of exosomes by macrophages. *P < 0.05, **P < 0.01 compared with the WT group; ##*P* < 0.01 compared with the MUT group.

Flow cytometry results exhibited that the M1/M2 ratio in the MUT group was lower than that in the WT group, whereas the M1/M2 ratios in the pcDNA3.1-WTAP + WT and pcDNA3.1-WTAP + MUT groups were higher than those in the WT and MUT groups, respectively (**Figures 9A, B**). As observed in ELISA results, IL-6 and TNF-α levels in the supernatant of macrophages co-cultured with RA-FLSs were elevated in the pcDNA3.1-WTAP + MUT group compared to the MUT group, with reduced levels of IL-10 and IL-13 (**Figures 9C-F**).

### 3.14 WTAP overexpression lowers circ-CBLB expression in macrophages

RT-qPCR results unveiled higher circ-CBLB expression in the MUT group than in the WT group but lower circ-CBLB expression in the pcDNA3.1-WTAP + WT and pcDNA3.1-WTAP + MUT groups than in the WT and MUT groups, respectively (**Figure 9G**). Fluorescence microscopy images validated the uptake of exosomes by macrophages (**Figure 9H**).

## 4 Discussion

The pathogenesis of RA is profoundly influenced by FLSs and macrophages, and macrophage polarization is a core mechanism in the development of RA (30). Macrophages can internalize exosomes via phagocytosis (31), while exosomes are essential in intercellular communication (32). In our study, exosomes were successfully isolated from FLSs and TNF-α-stimulated FLSs, which was validated by the identification of exosome-specific marker proteins CD63 and CD81 (33). Furthermore, the uptake of exosomes by macrophages was observed under the fluorescence microscope after specific staining with PKH26. In addition, TNF-α stimulation enhanced CD86 expression and increased the M1/M2 ratio in macrophages co-cultured with RA-FLSs, implicating that more macrophages are polarized toward the M1 phenotype in the inflammatory microenvironment of RA. To probe whether exosomes affected macrophage polarization, we used GW4869 to inhibit exosome secretion and then tested CD86 and CD206 expression. The results unraveled that exosome inhibition elevated the M1/M2 ratio in the RA-FLS + M0 + TNF-α and RA-FLS + M0 groups, which was supported by the levels of pro-inflammatory cytokines (IL-6 and TNF-α) and anti-inflammatory cytokines (IL-10 and IL-13). Fluorescence microscopy images exhibited a substantial reduction in exosomes internalized by macrophages after the addition of GW4869. Together, these results underscore that RA-FLS-derived exosomes exert repressive effects on macrophage polarization toward the M1 phenotype.

Exosomes play different roles in macrophage polarization. For instance, Fang et al. found that RA-FLS-secreted exosomes delivered cysteine dioxygenase 1 to drive M1 polarization of macrophages (34). Tang et al. observed that exosomes derived from ferroptotic renal tubular epithelial cells increased the production of M2 macrophages (35). Given that the function of exosomes is complex and the cargo of exosomes is altered in different cells (36), the present study further analyzed the contents in exosomes that affect macrophage polarization. Based on previous studies, we speculated that circ-CBLB was contained in the exosomes isolated in this study, which was validated by RT-qPCR detection of circ-CBLB expression in RA-FLSs, exosomes, and macrophages.The m^6^A modification level of circ-CBLB in RA-FLSs was examined with MeRIP-qPCR, which showed that the m^6^A modification level of circ-CBLB was prominently increased after TNF-α stimulation, illustrating that the inflammatory microenvironment of RA promotes the m^6^A modification level of circ-CBLB. Accumulating studies have reported that m^6^A modification can regulate the stability and degradation of non-coding RNAs (37). Additionally, exosomes after TNF-α stimulation have been revealed to affect macrophage polarization shift *in vitro* (38). Accordingly, it can be concluded that RA-FLSs secrete exosomal circ-CBLB and may degrade circ-CBLB in a m^6^A modification-dependent manner, thereby modulating macrophage polarization.

To explore the specific mechanism, we examined the expression of m^6^A modification-related key enzymes, such as METTL3, METTL14, WTAP, FTO, and ALKBH5. The results showed that the expression of METTL14, WTAP, and FTO in the RA-FLS + M0 + TNF-α group was significantly higher than that in the RA-FLS + M0 group. FTO is a demethylation gene. WTAP is the core regulatory subunit of m^6^A methyltransferase complexes, which not only catalyzes the activity of methyltransferases but also plays a key role in the recruitment and localization of METTL3 and METTL14 (39). Considering the function of WTAP and our experimental results, WTAP was selected as the next research object in our study. To verify whether WTAP protein binds specifically to circ-CBLB, we performed an RNA pull-down assay. The results demonstrated that WTAP protein mutually bound to circ-CBLB. Additionally, RIP-qPCR results displayed a marked increase in the enrichment of circ-CBLB in the IP group compared with the IgG group, further confirming the binding between WTAP protein and circ-CBLB. Despite no research on the association of WTAP with circ-CBLB, the critical role of WTAP in other diseases indicates that WTAP may regulate relevant biological processes by interacting with circRNAs. A former study documented that circ0003899 orchestrated the m^6^A modification of target mRNAs by binding to WTAP, thereby influencing the sensitivity of bladder cancer cells to cisplatin (40). In acute myeloid leukemia, WTAP can mediate the expression of circRNAs via m^6^A modification (41).

In this context, WTAP was silenced and overexpressed to investigate the specific mechanism by which WTAP mediated circ-CBLB expression through m^6^A modification in RA-FLSs. It was observed that WTAP overexpression obviously enhanced the m^6^A modification level of circ-CBLB and declined circ-CBLB expression in RA-FLSs, whilst WTAP silencing resulted in opposite trends. The actinomycin D assay exhibited that WTAP mediated circ-CBLB expression by accelerating its degradation. Meanwhile, the results of macrophage polarization markers demonstrated that WTAP overexpression prominently potentiated macrophage polarization toward the M1 phenotype. Subsequent rescue experiments substantiated the critical function of WTAP in regulating circ-CBLB expression and macrophage polarization. In the rescue experiments, co-transfection with the pcDNA3.1-WTAP overexpression plasmid significantly increased the M1/M2 ratio in both WT and MUT groups. The changes in downstream markers aligned with the macrophage polarization pattern, demonstrating elevated levels of pro-inflammatory cytokines (IL-6, TNF-α) and reduced levels of anti-inflammatory cytokines (IL-10, IL-13). When combined with previous experimental data, these results confirm that WTAP inhibits circ-CBLB expression by mediating its m^6^A modification, thereby influencing macrophage polarization. This mechanism ultimately induces an imbalance in pro-inflammatory and anti-inflammatory cytokines, contributing to the pathological progression of rheumatoid arthritis.

**Figure 10:**
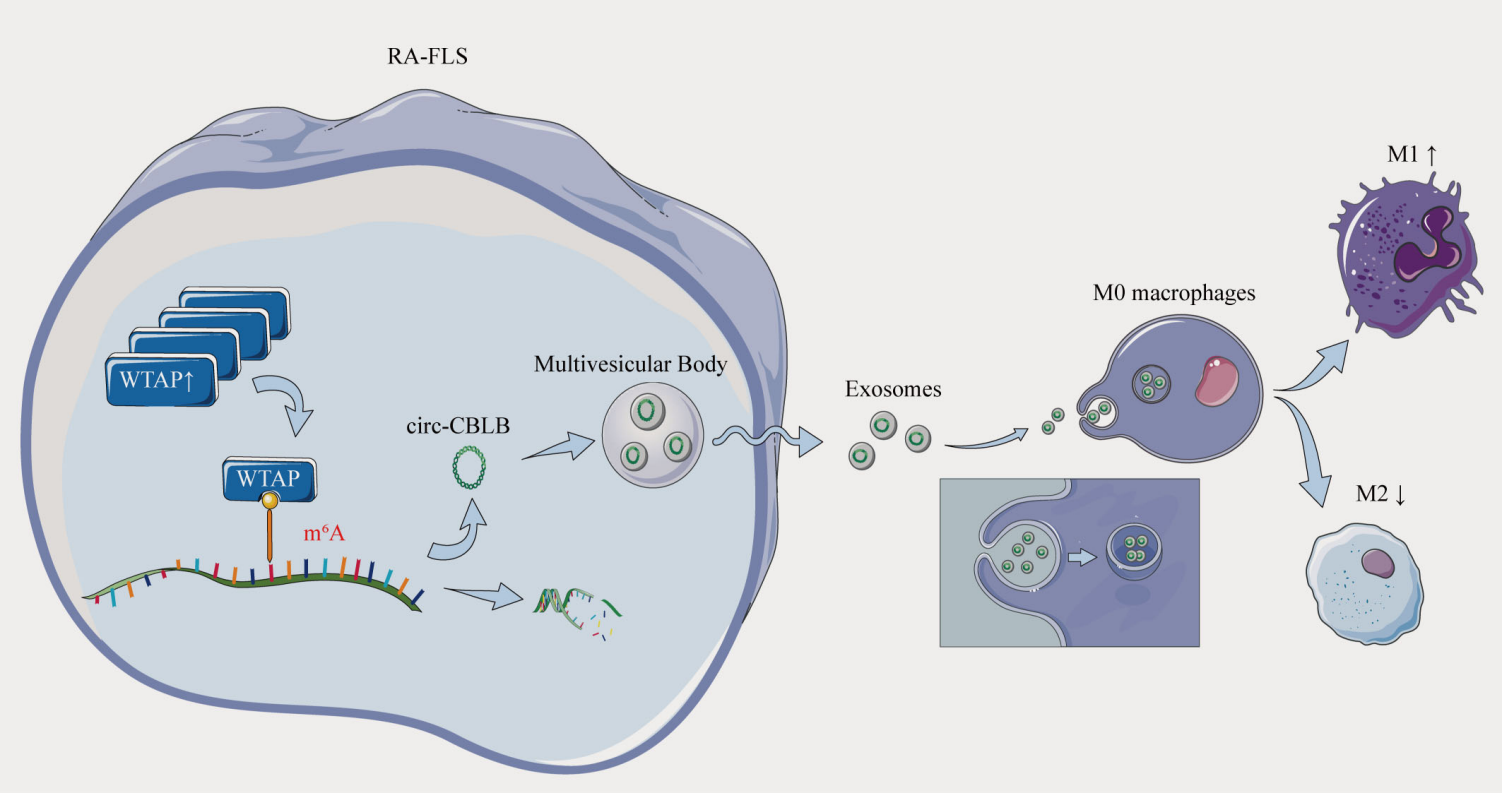
Overexpression of the m^6^A methylase WTAP stimulates polarization of undifferentiated macrophages towards the M1 phenotype by driving RNA methylation of exosomal circ-CBLB.

In conclusion, our study unraveled that exosomal circ-CBLB derived from RA-FLSs was involved in the occurrence and development of RA by modulating macrophage polarization via WTAP-mediated m^6^A modification (**Figure 10**). However, the study mainly involved *in vitro* experiments and did not explore therapeutic strategies targeting WTAP and exosomal circ-CBLB. Hence, further studies integrating animal experiments and clinical trials are warranted to delve into potential therapeutic approaches targeting WTAP or circ-CBLB, providing new ideas for the clinical treatment of RA.

## Data Availability

The original contributions presented in the study are included in the article/**Supplementary Material**. Further inquiries can be directed to the corresponding author.
