# Supplementary material for: WTAP modulates macrophage polarization in rheumatoid arthritis by targeting exosomal circ-CBLB via m6A modification
Source: Front Immunol. 2025 Jun 10;16:1601259. doi: 10.3389/fimmu.2025.1601259 (PMC12185438; doi:10.3389/fimmu.2025.1601259)
Supplement: Supplementary file 1 [file Table1.docx]

**Table 1** Sequences of the primers

| **Type** | **Amplicon Size（bp）** | **Forward primer(5'→3')** | **Reverse primer(5'→3')** |
| --- | --- | --- | --- |
| Hu-β-actin | 96 | CCCTGGAGAAGAGCTACGAG | GGAAGGAAGGCTGGAAGAGT |
| Hu-circ-CBLB | 133 | TCAGCTTCCTCATGTTCAGGT | TGCTAACGGACCAGTACACTT |
| Hu-Mettl3 | 184 | GCCTTCTGAACCAACAGTCC | CTGGCTTTCATGCACTCCTC |
| Hu-Mettl14 | 132 | TGCCTGTGATGGGTCCTTAG | ACAGGTGCCTATGCCATGTA |
| Hu-WTAP | 163 | AAGCAACAACAGCAGGAGTC | TCGCTGGGTCTACCATTGTT |
| Hu-FTO | 148 | AGACACCTGGTTTGGCGATA | GTTCCTGTTGAGCACTCTGC |
| Hu-ALKBH5 | 96 | TCTGCACTTGGTTGAGGTCT | AGGGTGTTTGCATGAGCTTG |

**Table 2** information of Antibody

| **Antibody** | **Manufacturer** | **Catalog Number** | **Lot Number** | **Dilution Ratio** |
| --- | --- | --- | --- | --- |
| Goat Anti-Rabbit IgG | Zs-BIO,China | ZB-2301 | 139931 | 1:10000 |
| CD63 | Affinity,China | AF5117 | 78p9380 | 1:1000 |
| CD81 | Affinity,China | DF2306 | 45H631 | 1:500 |
| β-actin | Zs-BIO,China | TA-09 | 19AW0505 | 1:1000 |
| Goat Anti-Mouse IgG | Zs-BIO,China | ZB-2305 | 142637 | 1:10000 |
| Goat Anti-Rabbit IgG | Zs-BIO,China | ZB-2301 | 139931 | 1:10000 |
| Mettl3 | Affinity,China | DF12020 | 45s7173 | 1:500 |
| Mettl14 | Abclonal,China | A8530 | 5500001847 | 1:500 |
| WTAP | Affinity,China | DF3282 | 88s1101 | 1:500 |
| FTO | Affinity,China | DF8421 | 21j3698 | 1:1000 |
| ALKBH5 | Affinity,China | DF2585 | 52e1437 | 1:500 |
| Cy3-labeled Goat Anti-Rabbit IgG (H+L) | Biosharp,China | BL058A | 23157201 | 1:400 |
| METTL3 | HUABIO,China | HA720002 | HO1008 | 1:100 |
| METTL14 | Abclonal,China | A8530 | 5500038191 | 1:100 |
| WTAP | Affinity,China | DF3282 | 88s1101 | 1:200 |
| ALKBH5 | Proteintech,USA | 16837-1-AP | 00136538 | 1:200 |
| FTO | ZENBIO,China | R24361 | N30AP9P | 1:100 |
